# Comparison of General Use of Antibiotics between Medical and Nonmedical University Students of Lahore

**DOI:** 10.1155/2023/8534944

**Published:** 2023-04-24

**Authors:** Fiza Ayub, Tahir Mehmood Khan, Muhammad Usman Amin, Mirza Rafi Baig, Allah Bukhsh, Khadija Zaman, Asma Afzal, Sana Bibi, Muhammad Umar Javed, Fouzia Naheed, Jamshaid Alam, Hafiz Ishfaq Ahmad

**Affiliations:** ^1^Institute of Pharmaceutical Sciences, University of Veterinary and Animal Sciences, Lahore, Pakistan; ^2^Department of Pharmacy, Abasyn University, Peshawar, Pakistan; ^3^Department of Clinical Pharmacy & Pharmacotherapeutics, Dubai Pharmacy College for Girls, Al Mizhar Dubai, UAE; ^4^Basic Health Unit, Tehsil Chichawatni, District Sahiwal, Pakistan; ^5^Rural Health Center, Basirpur, Tehsil Depalpur District Okara, Punjab, Pakistan; ^6^Rural Health Center Khayaban-E-Sir Syed Rawalpindi, Pakistan; ^7^Department of Animal Breeding and Genetics, Faculty of Veterinary and Animal Sciences, The Islamia University of Bahawalpur, Bahawalpur, Pakistan

## Abstract

**Objective:**

To compare the knowledge of antibiotic resistance between medical and nonmedical university students of Lahore. *Methodology*. An observational cross-sectional survey-based study was conducted among students of Lahore, Pakistan, from November 12, 2021, to December 13, 2021. The convenience sampling method was used to select students. Descriptive analysis and chi-square test were performed using Statistical Package for Social Sciences version 25.0.

**Results:**

52.9% medical and 42.25% nonmedical students knew about antibiotics. 24.1% medical and 18.3% nonmedical students do not take antibiotics without a prescription. 40.6% medical and only 19.3% nonmedical students knew about the course of antibiotics. Medical students let the minor ailments recover naturally compared to nonmedical students who visit the doctor more often. Both groups complete the course of antibiotics without a significant difference. 49% medical and 27.9% nonmedical students knew that bacteria can develop resistance against antibiotics. Most nonmedical students responded that antibiotics can work even after resistance. Medical students have better knowledge about the relationship of resistance with overuse and misuse.

**Conclusion:**

The knowledge of antibiotics and compliance to therapy of the nonmedical students were less than those of the medical students. Medical students were aware of the pattern of taking antibiotics because of their educational background. There is a dire need for awareness regarding antibiotic use in this group to conserve treatment options for future use.

## 1. Introduction

Humanity's struggle against infectious diseases is well known. But the discovery of antibiotics helped optimize human health by reducing the incidence of infections such as cholera, tuberculosis, smallpox, diphtheria, typhoid fever, syphilis, pneumonia, plaque, and typhus [1]. The era of antibiotics started after the discovery of penicillin by Alexander Fleming in 1928. Since 1940, antibiotics have been used widely in human and veterinary medicines [2]. This golden era of antibiotics witnessed the discovery of novel antibiotics and new classes of antibiotics, which helped increase the average life span of human life. Later, it was limited only to modifying existing antibiotics [1].

Some bacteria have become resistant to all the antimicrobials and are known as superbugs or pan-resistant organisms. It is alarming that multiresistant and pan-resistant bacteria are spreading rapidly worldwide, and infections caused by them are untreatable by any of the current antimicrobials. This ineffectiveness of the most recent and effective antibiotics can lead to an era where antibiotics will not be effective, and people will die from minor infections.

Globally, it is observed that the most common bacterial infections such as sepsis, urinary tract infection, and sexually transmitted infections are treated with antibiotics that have served to raise antibiotic resistance, which demonstrates that we are running out of antibiotics. The resistance to ciprofloxacin, an antibiotic used to treat urinary tract infections, has risen to 92.9% from 8.4%, showing severe antibiotic resistance threats.

Carbapenems, the last resort for *Klebsiella pneumonia*, are now observed to become resistant globally, and in most countries, the infections caused by *K. pneumonia* are left untreated. This has given rise to the spread of fatal infections worldwide.

Colistin is now considered the last resort for treating the infection caused by carbapenem-resistant Enterobacteriaceae. In most countries, bacterial species resistant to colistin have been spotted. This resistance has given rise to the spread of life-threatening and alarming situations.

According to a 2018 study conducted by the World Health Organization (WHO), more than half of a million cases of rifampicin-resistant tuberculosis were reported against which most of the anti-TB drugs are resistant. This drug resistance has given a surge to more severe cases of TB all over the world.

Over the last two decades, many studies have been published indicating the worsening situation of antimicrobial resistance in Pakistan*. E.coli* resistance against cephalosporin, particularly ceftriaxone, has increased from 18% in 2017 to 94% in 2018*. E.coli* resistance has also increased against carbapenems: for imipenem to 15% from 10%, ertapenem to 29% from 23%, and meropenem to 20% from 19%.

*Acinetobacter baumannii* (*A. baumannii*) is also developing resistance against quinolones and aminoglycosides. *A. baumannii* is reported to develop 60% resistance against carbapenems. Ceftriaxone resistance against XDR typhoid increased to 29.11% in 2018 from 18% in 2017. *S. aureus* has developed 68% resistance to cefoxitin but remains susceptible to vancomycin.

This shortage of effective antibiotics is equally alarming for both developed and developing countries. It will take the bar on the patient by increasing their hospital stay and the cost of the treatment [3, 4].

All the students (medical and nonmedical) should be aware of the emerging antibiotic resistance problem to tackle antimicrobial resistance adequately in the future. This study is aimed at comparing the practice of antibiotics and knowledge of resistance in medical and nonmedical university students in Lahore, Pakistan.

## 2. Material and Methods

### 2.1. Study Population

#### 2.1.1. Study Design and Population

It was conducted as a cross-sectional study on the students of University of Veterinary and Animal Sciences Lahore, University of Engineering and Technology, Lahore, and Government College of University Lahore from November 27, 2021, to December 10, 2021. Convenient sampling was used for data collection. The minimum sample size should be 384, with a 95% confidence interval, a 5% margin of error, and a prevalence of 50%; *d* is 0.05 as calculated by the formula [5].

For 95% CI, the value of *Z* is 1.96.

(1) $$\begin{array}{c} n=\frac{Z^{2}P\left(1-P\right)}{d^{²}} \end{array}$$

where *n* is the sample size, *Z* is the *Z* statistic for a level of confidence, *P* is the expected prevalence or proportion (in proportion of one; if 20%, *P* = 0.2), and *d* is the precision (in proportion of one; if 5%, *d* = 0.05).

The eligibility criteria of our study were no faculty member or ancillary staff and not enrolled in any university of Lahore and age of students must be 17–30 years.

#### 2.1.2. Data Collection

The data were collected through Google Forms composed of 15 questions. It is further divided into 2 sections.  Section 1 includes the demographic data which were related to age, gender, education, and qualification.  Section 2 includes the questions related to antibiotics to assess the respondents' knowledge which includes the common antibiotics they have heard of, their pattern of taking antibiotics, and what they do to leftover antibiotics. The last questions were about their knowledge regarding antibiotic resistance, what they know about antibiotic's working pattern after the bacteria are resistant to the antibiotic, and what could be the possible reasons of antibiotic resistance. We forwarded the Google Form online; 40% of the students did not respond to the form.

The 10 questions were designed using *the Antimicrobial Resistance Module for Population-Based Surveys* (*2008*), including whether bacteria develop resistance and the possible reasons for antibiotic resistance.

#### 2.1.3. Data Analysis

The data was analyzed using Statistical Package for the Social Sciences (SPSS) 25. Nominal variables, including questions related to knowledge of antibiotics, were analyzed by calculating percentages and frequencies. Descriptive statistics were used to analyze the continuous variables, e.g., age and gender. A chi-square (level of significance, *p* < 0.05) test was applied to the data to compare the knowledge of medical and nonmedical students.

#### 2.1.4. Ethics Approval

The ethical approval to conduct this study was taken from the Institute of Pharmaceutical Sciences (IPS) at the University of Veterinary and Animal Sciences, Lahore. The study purpose was informed to the patients clearly before collecting information. An application for ethical approval was designed and forwarded to the Ethical Review Board (ERB) to explain the unobjectionable nature and objectives of this research study. The study protocol was approved by the institutional review board of the University of the Veterinary and Animal Sciences Lahore (Ref: IPS-UVAS 2022/114).

## 3. Results

### 3.1. Attributes of Participants

A total of 684 university students participated in our research, including 308 (45%) males and 376 (55%) females, of which 364 (53.1%) were medical students and 321 (46.9%) were nonmedical students. Among these respondents, 504 (73.6%) were undergraduates, 128 (18.7%) were university graduates, 49 (7.2%) were postgraduates, and 4 (0.6%) were postdoctorates (Table 1).

### 3.2. Antibiotic Resistance Knowledge

The majority of participants, 651 (95.2%), were aware of the term antibiotics. Out of these, only 526 (76.9%) knew about bacterial resistance developed by antibiotics. A total of 501 (73.2%) knew that antibiotics would not remain effective after bacteria develop resistance. On the other hand, 183 (26.8%) responded that antibiotics would remain effective even after resistance.

In the questionnaire, question numbers 13, 14, and 15 were designed to ask respondents about the possible reason for antibiotic resistance. Their response was excessive use, not completing the course of antibiotics, not linked to the way of use, and any other by 59.9%, 46%, 9.3%, and 12.3% of respondents, respectively.

The medical students were more compliant toward course completion. Medical students were well aware that bacteria can develop resistance to antibiotics and that antibiotics will not be effective once bacteria are resistant. After analyzing the findings via chi-square, it was observed that the *p* value of all the variables mentioned above is <0.001, which demonstrates that alternative hypotheses are accepted (Table 2).

The knowledge about commonly used antibiotics in Pakistan, i.e., Augmentin (amoxicillin), Novidat (ciprofloxacin), Amoxil (metronidazole), and Flagyl, was also assessed. The majority of our study population, 520 (75.8%), were aware of Augmentin, 505 (73.6%) knew about Flagyl, 428 (62.4%) knew about Amoxil, and 340 (49.6%) were aware of Novidat. The majority of respondents, 394 (57.6%), used antibiotics without a prescription. In this study population, 410 (59.9%) were aware of pattern of taking the antibiotics (Table 3).

## 4. Discussion

This study was aimed at comparing knowledge and usage of antibiotics between medical and nonmedical university students of Lahore. According to the findings of this study, 20% of 364 medical students and 52% of 320 nonmedical students responded that antibiotics would remain effective even when the bacteria are resistant. Still, clinically, bacteria are becoming multidrug resistant, causing a shortage of treatment options for multidrug resistance bacteria. A substantial increase in antibiotic resistance is becoming a worldwide challenge because of the dissemination of resistant microorganisms at the community level.

A study conducted in Pakistan shows the major reasons for lack of awareness and inappropriate use of antibiotics. Easy availability of antibiotics without prescription, self-medication with antibiotics, cessation of therapy without course completion, and lack of counseling regarding antibiotic use by healthcare professionals are some major reasons. Low health literacy among students (especially nonmedical students) can lead to improper use of antibiotics. As per the findings of this research, 75% of the students are well aware of Augmentin, which is one of the most commonly used antibiotics in Pakistan. Most of the students are only aware of the names of the antibiotics but not the appropriate usage of them. Surprisingly, the population labeled some drugs such as Panadol (paracetamol), Risek (omeprazole), Rigix (cetirizine), Methycobal (methylcobalamin), and Surbex z (food supplements) as antibiotics. According to responses we had from our populations, most of the people misapprehended Panadol (paracetamol) and different antiallergics as antibiotics.

Only 1% of medical students and 5% of nonmedical students responded that bacteria could not develop resistance, while 6% and 35% were unsure about the occurrence of resistance, respectively.

Nonadherence to antibiotic therapy was reported by 42% of nonmedical students and 28% of medical students. According to lab findings of a study published in 2016, the risk of increased regrowth and bacterial survival possibilities was augmented due to the extension of the lag phase upon removal of antibiotics. These findings are crucial from a clinical perspective because of resistance and disease recurrence. Quinolones and tetracycline are most susceptible to resistance via this mechanism [6]. A study including 200 adults was conducted in 2016 in Karachi, Pakistan, to assess the irrational use of antibiotics. Results indicated that 17% of their population did not follow the course of antibiotics [7]. As per the findings of the current study, 65.2% of the 684 individuals reported that they follow the course of antibiotics.

According to a study conducted in India, the antibiotic resistance knowledge of medical students was assessed. They received 474 total responses from 103 medical colleges. The mean knowledge score was calculated to be 4.36 ± 0.39. This knowledge was much higher than the knowledge of first-year students. Almost 45% of the total population acknowledged that they take antibiotics without a doctor's prescription. Conclusively, the knowledge about antibiotic resistance was almost acceptable, but they needed to improve their attitude and practices [8].

## 5. Strengths and Weaknesses

The data collected for this research study was through a self-administered questionnaire, and the data was not skewed. The minimum sample size required for this study was 384, but we approached 684 respondents to reduce error and enhance the authenticity of our result. The major drawback of this study is that we collected the data at a small level that otherwise would have been collected at the provincial level. Another downside of this research is convenience sampling.

## 6. Conclusion

The current study concludes that university students of Lahore are mindful of antibiotic usage. Still, there is a dire need to create awareness, particularly among nonmedical students, about antibiotics to limit the shortage of treatment options in the future. There is an appalling need for awareness campaigns and seminars for students to enhance their knowledge about antibiotic use. Future research can involve participants from various educational backgrounds, such as zoology and botany.

## Figures and Tables

**Table 1:** Population demographics.

Variables	*N* (%)
Gender	
Female	55.0
Male	45.0
Education	
Medical	53.1
Nonmedical	46.9
Qualification	
Undergraduate	73.6
Graduate	18.7
Postgraduate	7.2
Postdoctorate	0.6

**Table 2:** Variables of the population compared.

Variables	Categories		*p* value	df	*X* ^2^ value
	Medical	Nonmedical			
Do they complete a course of antibiotics?					
Continue your therapy for further 3 days	261 (38.2%)	185 (37%)	<0.001	1	14.483*a*
Quit the therapy because you are fine	103 (15.1%)	135 (19.7%)	—		
Can bacteria develop resistance?					
Yes	335 (49%)	119 (27.9%)	<0.001	2	100.944*a*
No	6 (0.8%)	17 (2.6%)	—		
Maybe	23 (3.4%)	112 (16.3%)	—		
Can antibiotics work after bacteria develop resistance?					
Yes	73 (10.7%)	110 (16.1%)	<0.001	1	17.820*a*
No	291 (42.5%)	210 (30.7%)	—		

**Table 3:** Variables included in the study.

Variables	Categories	Frequencies	
		Medical	Nonmedical
Do they know about antibiotics?	Yes	362 (52.9%)	289 (42.25%)
	No	2 (0.29%)	31 (4.5%)

Antibiotics they have already heard	Augmentin	302 (44.1%)	218 (31.7%)
	Novidat	210 (30.7%)	130 (18.9%)
	Amoxil	254 (37.1%)	174 (25.3%)
	Flagyl	272 (39.7%)	233 (33.9%)
	None	8 (1.1%)	24 (14.5%)

Do they take antibiotics without a prescription?	Yes	119 (17.4%)	111 (16.2%)
	No	165 (24.1%)	125 (18.3%)
	Maybe	76 (11.1%)	80 (11.7%)
	Always	4 (0.6%)	4 (0.6%)

Do they know about the course of antibiotics?	Yes	278 (40.6%)	132 (19.3%)
	No	86 (12.6%)	188 (27.5%)

How do they respond to disease?	Let it recover naturally	170 (24.8%)	106 (15.4%)
	Visited the doctor for recovery	145 (21.15%)	145 (21.15%)
	Buy from the pharmacy	96 (14%)	107 (15.6%)
	Use randomly suggested medicines	18 (2.6%)	27 (4.0%)

What do they do to leftover antibiotics?	Saved for personal future use	222 (32.5%)	204 (29.6%)
	Give to someone else	16 (2.3%)	41 (6%)
	Taken back to the pharmacy	62 (9.1%)	41 (5.9%)
	Throw away	96 (14%)	74 (14.8%)

Do they complete a course of antibiotics?	Continue your therapy for further three days	261 (38.2%)	185 (37%)
	Quit the therapy because you are fine	103 (15.1%)	135 (19.7%)

Can bacteria develop resistance?	Yes	335 (49%)	119 (27.9%)
	No	6 (0.8%)	17 (2.6%)
	Maybe	23 (3.4%)	112 (16.3%)

Can antibiotics work after bacteria develop resistance?	Yes	73 (10.7%)	110 (16.1%)
	No	291 (42.5%)	210 (30.7%)

What is the reason for resistance according to them?	Excessive use	237 (34.6%)	173 (25.3%)
	Not completing the course on antibiotic	202 (29.5%)	115 (16.5%)
	It is not linked to the way we use antibiotics	33 (4.65%)	33 (4.65%)
	Any other	28 (4.1%)	56 (8.2%)

## Data Availability

The data used to support the findings of this study are included within the article.
